# Unraveling In vivo Potential of Curcumin-loaded Graphene Quantum Dots on Drug Delivery and Release Kinetics Aspects of Cancer Treatment

**DOI:** 10.7150/ntno.96559

**Published:** 2024-08-13

**Authors:** Mochamad Z. Fahmi, Siti F. A. Sugito, Aswandi Wibrianto, Siska Novania, Shinta Widyastuti, Musbahu Adam Ahmad, Satya C. W. Sakti, Lee H. Voon

**Affiliations:** 1 Department of Chemistry, University Airlangga, Surabaya 60115, Indonesia.; 2 Supra Modification Nano-Micro Engineering Research Group, Universitas Airlangga, Surabaya 60115, Indonesia.; 3 Nanotechnology and Catalysis Research Centre (NANOCAT), Universiti Malaya, 50603, Kuala Lumpur, Malaysia.

**Keywords:** Graphene Quantum Dots, curcumin, anti-cancer, health, therapy

## Abstract

This study introduces an innovative magnetic-based multifunctional anti-cancer drug carrier aiming to enhance the efficacy of curcumin in cancer therapy. The research investigates the potential of Graphene Quantum Dots (GQDs) as a curcumin drug delivery system for inhibiting *in vivo* cancer growth. GQDs with a particle diameter below 10 nm were synthesized via hydrothermal and Hummers methods, exhibiting homogeneity and crystalline structure according to AFM and XRD analyses. FTIR analysis confirmed functionalization success, revealing the formation of bonds between GQDs and curcumin. The optical properties of GQDs were assessed using a UV-Vis spectrophotometer and spectrofluorometer, resulting in vigorous fluorescence with a quantum yield of 1.32%. Subsequently, loading curcumin onto GQDs (CQDs/cur) resulted in an efficient system for delivering the anti-cancer drug, demonstrating significant *in vivo* efficacy. It was indicated by reduced tumor diameter and increased body weight in mice. Furthermore, the release kinetics of curcumin from GQDs were analyzed using the Peppas-Sahlin equation under varying pH conditions (4, 7, and 9), revealing the highest release rate in acidic conditions. In conclusion, this study highlights the potential of GQDs as highly efficient carriers for targeted curcumin delivery, showcasing promising prospects in cancer treatment.

## Introduction

Graphene Quantum dots (GQDs) as graphene sheet-based nanomaterials with sizes <50 nm has been widely developed and utilized by researchers in various aspects such as solar cells 1, photodetector 2, photo-electrocatalytic 3, fluorescent agent 4, LEDs 5, batteries 6, sensors 7, bioimaging 8, and drug carriers 9. As a new type of nanoscale material, GQDs have potential applications in biomedicine and health sectors as health instruments and products. GQDs mainly comprise organic compounds known for their non-toxic nature and other promising properties. They are hydrophilic, highly biocompatible, inert, and can dissolve easily in water due to their large surface area. Hence, they can be utilized in drug delivery and medical diagnostic probes. The advantages of GQDs have led to significant advancements in medical applications, particularly in cancer therapy and diagnosis. Chemotherapy, the most common cancer treatment, has historically been expensive and complicated, with many drawbacks and adverse side effects that impact the sufferer's quality of life. Although GQDS have a low-toxic feature, they are limited in directly injuring cancer cells. Therefore, many reports suggest modifying this material to deliver active drug agents to cancer cells. Combining GQDs with several drugs could be a practical approach to address these problems.

The development of natural products has shown more promise in terms of safety and eligibility on cancer treatment. Curcumin is an organic compound commonly used in cancer therapy due to its proven therapeutic benefits. It has antioxidant and anti-inflammatory effects and is low in toxicity when introduced into the body. It can specifically target cancer cells by regulating multiple cell signaling pathways 10-13. Therefore, GQDs nanoparticles can potentially enhance the efficacy of curcumin as an anti-cancer agent. In previous studies, there were issues with the pH condition at the end of the experiment, which was often too acidic due to the synthesis process being carried out in an acidic environment. As a solution, other compounds were introduced into the mixture to neutralize the excessive acidity of the final product. Several compounds/elements were incorporated into the structure of GQDs in order to favor loading of curcumin on the GQDs structure. The compounds/elements tested for doping with GQDs include glucosamine 9, Tryptophan 14, Metal-Organic 15, Iron 16, and heteroatom elements (N, S, and F) 17. However, one challenge faced due to doping is that the synthesis time increases. Furthermore, to prevent imminent acid precitation in the synthesis solution, Human Serum Albumin (HSA) had to be added 4. Additionally, curcurmin is generally insoluble in water. Such solubility limitation makes its preparation complex. The hydrophobic nature of the compound further limits its application in body conditions. Fortunately, loading curcumin to GQDs improves water solubility, making it more readily applicable to the body 18. However, it is necessary to test the safety and efficacy of GQDs/cur as a drug delivery system for curcumin before consumption 19.

Researchers are already formulating methods of loading GQDs with curcumin for anticancer application. GQDs-Fe_3_O_4_-FA was synthesized via a one-step co-precipitation process for pH-responsive and targeted delivery of curcumin; however, the system exhibits hydrophobicity 20. Trp-GQDs were fabricated through a facile oxidation method and loaded with curcumin as a hydrophobic anticancer agent, but *in vivo* results are lacking to support health applications 9. Cur/GQDs and Cur/GlcN-GQDs were synthesized as anti-cancer agents, and the resulting nano-assembly was used for killing MCF-7 breast cancer cells with higher releases observed at an acidic condition. However, there was also a lack of *in vivo*data to support their use *in vivo* 14. To date, a number of *in vitro* studies on curcumin loaded with GQDs had been conducted. Most of the studies suggested that GQDs areeffective drug delivery systems for curcumin without causing excessive toxicity 19, 21. However, in addition to *in vitro* studies, it is crucial to evaluate the efficacy of GQDs/cur through *in vivo* investigation to assess the safety of GQDs/cur when applied to the body.

**Scheme 1:**
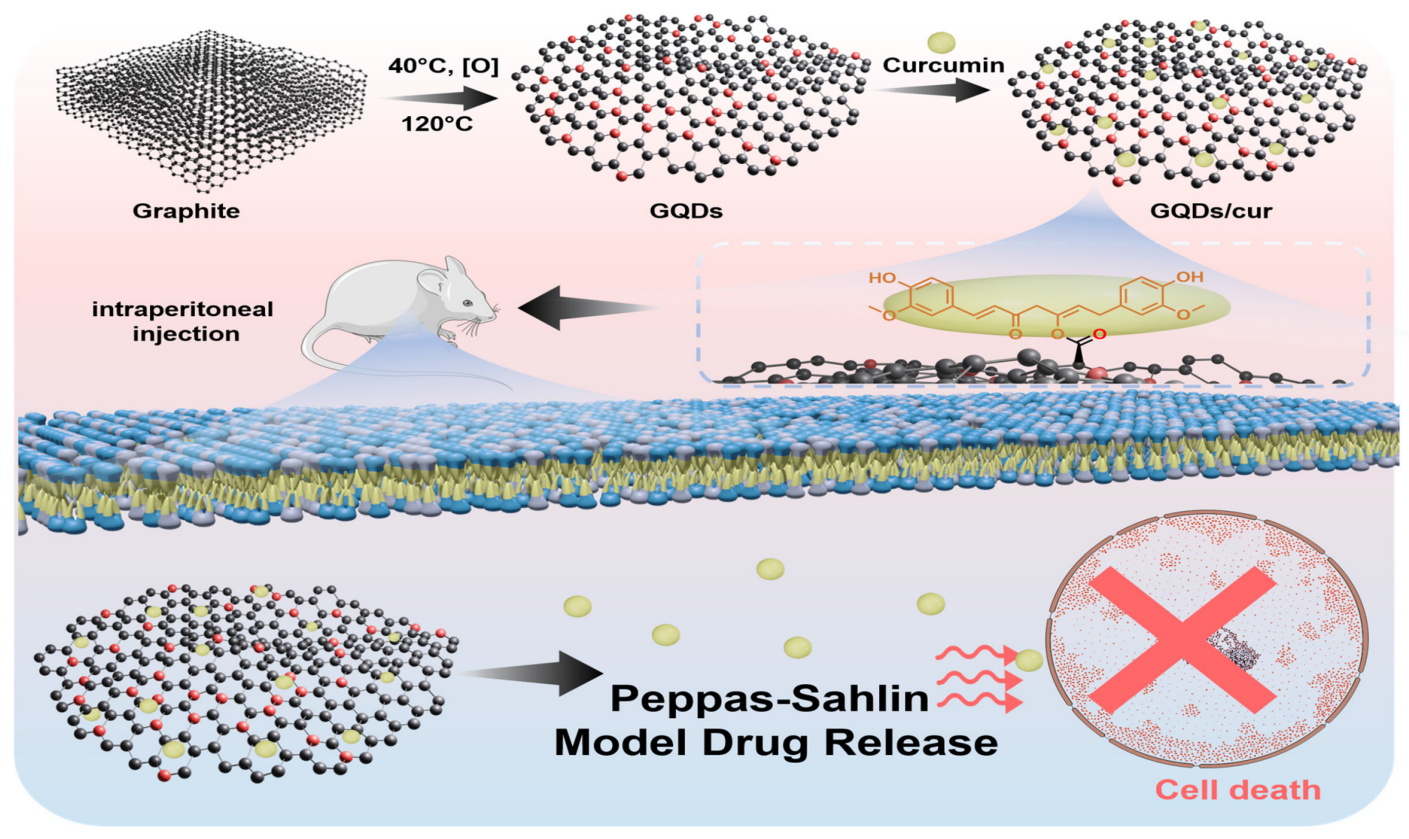
Schematic illustration the synthesis of GQDs/cur and curcumin release model after mice injection.

Moreover, release kinetic study is used to evaluate drug delivery system profile. This test assesses the drug's release profile and belongs to a crucial aspect of dosage formulation. It is particularly crucial for slow-release dosage formulations, focusing on controlling the drug release rate 22. With the information on the drug release profile, it is possible to know the rate of drug release and the time it takes for the drug to be released from the preparation. Previous studies have only employed first-order, pseudo-second-order, and Higuchi mathematical models to study drug release kinetics 9, 14, 22. However, observations of curcumin-loaded GQDs on treated cancer cells *in vivo* and the kinetic mechanism of curcumin release from GQDs have never been explored. Thus, the present study explores the synthesis and characterization of curcumin-loaded GQD. Further performance of the nanomaterial on treating cancer cells *in vivo* became a critical aspect pursued in this study (as shown on **Scheme 1**). The observation of release pattern of curcumin from GQDs was prioritized using various mathematical models, including zero order, first order, Kopcha, Higuchi, Hixson-Crowell, and Korsmeyer-Peppas. Finally, the drug delivery activity of GQDs/cur was investigated through various kinetics patterns, supported by an *in vivo* study for anti-cancer treatment.

## Experimental

### Materials

Pure graphite (C), Concentrated Sulfuric Acid (H_2_SO_4_), Sodium Nitrate (NaNO_3_), Potassium Permanganate (KMnO_4_), Anhydrous Sodium Carbonate (Na_2_CO_3_), Ethanol, EDTA, HSA (Human Serum Albumin) polymer, ethanol amine, Sodium Bicarbonate (NaHCO_3_), and distilled water. All the materials were purchased from Sigma Aldrich, Germany.

### Preparation of GQDs

GQDs were prepared by stirring 0.4 g graphite with 25 mL H_2_SO_4_ for 40 mins, then adding 10 g NaNO_3_ while stirring in an ice bath. Once the temperature drops to 0°C, add 0.75 g of KMnO_4_. Stir the mixture at 40°C for 40 minutes until it forms a black paste called GO. Next, stir the GO paste at 120°C for 12 hours. Then, add 125 mL of distilled water and Na_2_CO_3_ until it reaches a pH of 3. After 24 hours, filtrate the GO and dialyze it into a 100 nm active membrane for 24 hours. Additionally, the outer membrane solution was heated under an autoclave at 180°C for 120 hours, producing white GQDs using the hydrothermal method.

### Preparation of GQDs/cur

To prepare the solution, 0.5 grams of GQDs were dissolved in 30 milliliters of distilled water using ultrasonication for an hour. Then, a solution of curcumin and GQDs in a 1:10 ratio was added to the mixture while stirring, and the resulting mixture was left to stir for 24 hours until the GQDs/cur loading was completed.

### Characterization

UV-Vis absorption was acquired using UV-1800 UV-Vis Spectrophotometer (Shimadzu, Japan). Photoluminescence (PL) emission spectra were performed on PerkinElmer LS 55 spectrofluorometer with 20 kW xenon lamp. The FTIR spectra were measured on an Infrared (IR) Tracer-100 (Shimadzu, Japan). The crystallographic phase of Cu-CDs was determined by X-ray diffraction (XRD) pattern on Rigaku 18 kW rotating anode source X-ray diffractometer equipped with graphite monochromatized Cu K_α1_ line (λ=0.15405 nm) at a range from 10° to 90° and scanning speed of 4°/min (Rigaku, Japan). An atomic force microscope image was obtained by scanning probe AFM5500M instrument (Hitachi Co., Japan). Raman spectrum was analyzed on MRS-320 Raman Instrument system (Horiba Ltd., Japan).

### Colloidal stability

The optimization of GQDs was evaluated at different pH values and salt concentrations. First, the pH range of 3 to 12 was tested by adjusting with 1N HCl and 1N NaOH. The effect of salt on GQDs was then determined by adding 0.1, 0.2, 0.3, 0.4, 0.5 and 1 M NaCl to the mixture. The mixture was observed for 0, 6, 12, and 24 hours and then measured using a UV-Vis spectrophotometer and a turbidity meter.

### % Drug loading and efficiency

The loading process results in the formation of GQDs that bind with curcumin, referred to as GQDs/cur. This interaction is primarily due to π-π stacking bonds, facilitated by the larger molecular weight of GQDs compared to curcumin. The concentration ratio of GQDs to curcumin is 10:1, and **Equations (1)** and **(2)** can be used to determine the loading efficiency and loading amount 23.


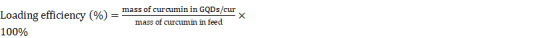

(1)


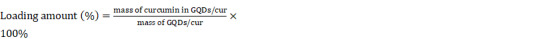

(2)

These calculations have determined that the loading efficiency of GQDs/cur is 50%, with a loading amount of 5.1%.

### Release of curcumin from GQDs/cur

In order to create a calibration curve for curcumin, a 100 ppm curcumin solution was prepared and then diluted to create standard solutions with concentrations of 2, 6, 10, 14, and 20 ppm. The calibration curve was established by measuring the absorbance of standard solutions at 430 nm to determine the concentration of released curcumin (**Figure 4a**). The GQDs/cur samples were loaded into an activated membrane and left for 2 hours. About 4 mL of the dialyzed samples were placed into a beaker with 50 mL of distilled water to release curcumin from the GQDs. The release profile was measured at 5, 10, 30, 60, 120, 180, 360, 540, 720, and 1440 minutes in buffer solutions with 4, 7, and 9 pH values, respectively. For each measurement, 3 mL of solution was replaced with another solution of the same volume. The solution was analyzed for absorbance using a UV-Vis spectrophotometer at the predetermined wavelength. The actual concentration of released curcumin was calculated using **Equation (3)**. The maximum wavelength to measure the released curcumin levels from GQDs was determined.


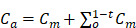

(3)

where *Ca* (μg/mL) represents the actual concentration at time *t(s)*, *Cm* ((μg/mL) signifies the measured concentration at time *t,v* and *V* demonstrate the volume of the DI water taken (mL) and the total volume of the DI water (mL), respectively.

### Kinetical study of released curcumin from GQDs/cur

Mathematical models such as zero order, first order, Peppas-Sahlin, Higuchi, Hixson Crowell, and Korsmeyer-Peppas will be used to evaluate the kinetics of curcumin release, as presented in **Equation (4)**, **(5)**, **(6)**, **(7)**, **(8)**, and **(9)**, respectively.


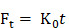

(4)

where F_t_ refers to released curcumin fraction in time *t*, and K_0_ indicates the zero order release constant (s^-1^).


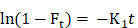

(5)

where K_1_ demonstrate the first order release constant (s^-1^).


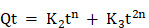

(6)

where Qt represents the quantity of drug released at time t, while K_2_ and K_3_ represent the Peppas-Sahlin constants 24, 25. The values of K_2_ and K_3_ determine the drug release mechanism within this model. The K_2_ outlines the process of controlled release by diffusion, while the K_3_ explains the polymer structure release, namely the relaxation of the polymer chain. If K_3_ surpasses K_2_, the main factor determining the drug release mechanism is Fickian diffusion. Meanwhile, if K_2_ is less than K_3_, the relaxation of the polymer chain plays an essential role in the drug release process.


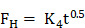

(7)

where F_H_ refers to the fraction of released curcumin in time t and K_4_ is Higuchi release constant (s^-0.5^).


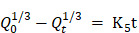

(8)

where *Q*_0_ represents the quantity of drug released at initial time t, and K_5_ refers to the Hixson crowell release constant (s^-1^).


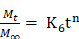

(9)

where the variables M_t_ and M_∞_ represent the quantities of curcumin released at time t and ∞, respectively. K6 denotes the Korsmeyer-Peppas release constant, whereas n represents the release exponent. The variable n represents Fickian diffusion. If the value of n ≤ 0.5, it indicates non-Fickian or anomaly occurrences. If the value of 0.5 < n < 1, it implies a lack of temporal dependence on release kinetics. In other words, it suggests zero-order kinetics.

### *In vivo* investigation

Male *Mus musculus* mice are acclimated to their cage environment for one week before being provided ad libitum feeding. The mice maintenance room temperature is 22 ± 3 ℃ with 12 hours of bright and dark lighting each. This study identified 16 mice per group by measuring their body weight and marking certain limbs. The mice were then fasted for 14 hours before treatment.

Benzopyrene compounds were used to create cancer cells in this research. Satiated mice were injected with 30mg of the carcinogen solution in 10 mL of liquid. Each mouse required 0.2mL of the solution for injection. All mice in each group were given benzopyrene orally every other day for a month to induce carcinoma formation in their bodies. Confirming the presence of cancer cells or tissues in mice involves palpation, which is essentially a process of touching the cancer nodules. After one month of benzopyrene induction, the mice are thoroughly examined by palpating their entire body to determine the presence of any nodules. The total number of nodules and their diameter are then recorded, and the presence of nodules indicates that the mice have cancer.

After discovering cancerous nodules in all mice samples, the research treatment will commence using the intraperitoneal injection method. The mice were divided into four groups for the injection procedure. The P0 group was injected with a 0.9% NaCl solution, the P1 group received a 0.2 mL curcumin solution, the P2 group was injected with 0.2 mL of GQDs, an anti-inflammatory agent, and the P3 group received a 0.2 mL solution containing both QDs and curcumin that were carried out every two days for one month, within twice a week, the mice were weighed to monitor changes in their body weight during treatment.

After treating the mice, it was necessary to monitor the progression of cancer cells until euthanasia. To assess the impact of the treatment, changes in body weight before euthanasia were observed. The euthanasia process involved nape-pulling, followed by dissection to measure the diameter of cancer cell nodules in relevant tissues and organs. We also collected various organs for toxicity testing.

### Ethical approval

Ethical clearance to conduct the present study was obtained from the Health Research Ethical Clearance Committee Faculty of Dental Medicine Universitas Airlangga (ethical clearance number: 157/HRECC.FODM/IV/2021).

## Results

### Characterization of GQDs

**Figure 1:**
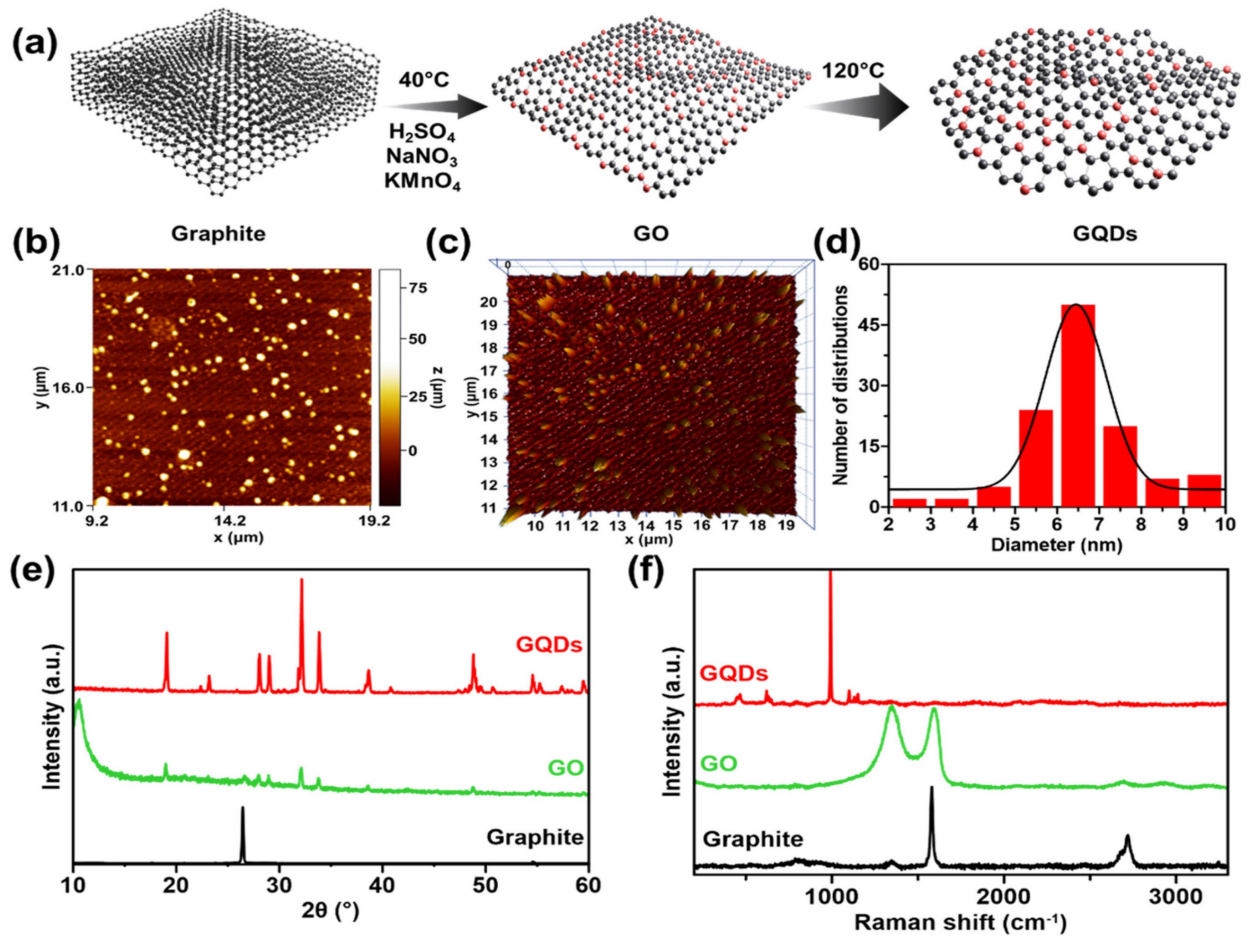
(a) Synthesis route representation of GQDs. Morphological properties of as-synthesized GQDs. (b) 2D and (c) 3D AFM images, supported with (d) particle size distribution of GQDs. (e) XRD patterns and (f) Raman spectra of initial graphite (black), GO (green), and GQDs (red).

GQDs were prepared. GO was first prepared from graphite reacted with H_2_SO_4_, NaNO_3_, and KMnO_4_. The resulting mixture was subjected to a hydrothermal process, producing white GQDs, as shown in **Figure 1a**. The molecular structure characteristics of synthesized GQDS can be obtained using XRD, AFM, and Raman spectroscopy. The process of characterization using AFM is aimed at determining the diameter size of a particle. It displays topographic information on the sample's surface in three dimensions with high resolution, providing a way to determine the size distribution of nanoparticles present on the sample surface.** Figures 1b-d** show a histogram of the particle size distribution for GQDs, which shows a relatively homogeneous distribution with an average diameter of approximately 6.44 ± 0.05 nm. In addition, it also provides a visual representation of the shape of the GQDs sample in both two and three dimensions. The 3D image reveals that the height of each particle varies significantly, with a spike-like shape. This condition indicates that the GQDs formed are nanoparticle-sized particles coated by graphene of less than 100 nm on the surface of the GQDs 26, 27. On the other hand, the diffractogram of the synthesized graphite and GQD is shown in **Figure 1e**. The figure displays an XRD graphite peak with a vigorous intensity at 2*θ* = 26.6 ° and a d-spacing of 3.37 Å. Additionally, a peak with a small intensity at 2*θ* = 54.6 ° and a d-spacing of 1.68 Å is also observed. These values follow the 2*θ* and d-spacing values of pure graphite 28, 29. The GO synthesized from graphite has peak characteristics that match JCPDS 47-0787 at 11°, 17°, and 21°, with a size of 12.82 nm using the Scherrer equation. In GQDs, several sharp peaks may indicate both peak and crystalline GQD samples. The GQDs produced seven peaks at 19.07°, 28.06°, 29.02°, 32.16°, 33.87°, 38.65°, and 48.84°. In order to investigate the crystallization, structural disorder, and defects of the GQDs, Raman measurements were performed at room temperature. In **Figure 1f**, the Raman spectrum of GQDs shows one peak at 988 cm^-1^. The second peaks are absent because the salt molecule used in synthesis is too large, covering the edges of the GQDs molecule and masking their spectra. The GO spectra exhibit two prominent peaks at 1349 and 1598 cm^-1^, corresponding to D and G bands, respectively 30, 31. The G band indicates the presence of sp^2^ carbon bonds, whereas the D band signifies defects or irregularities at the edges of the GO sample. The graphite spectra display two distinct peaks. The first peak is the G band, which appears at 1592 cm^-1^. The second peak is the 2D band located at 2723 cm^-1^ and 2681 cm^-1^, as a shoulder peak.

**Figure 2:**
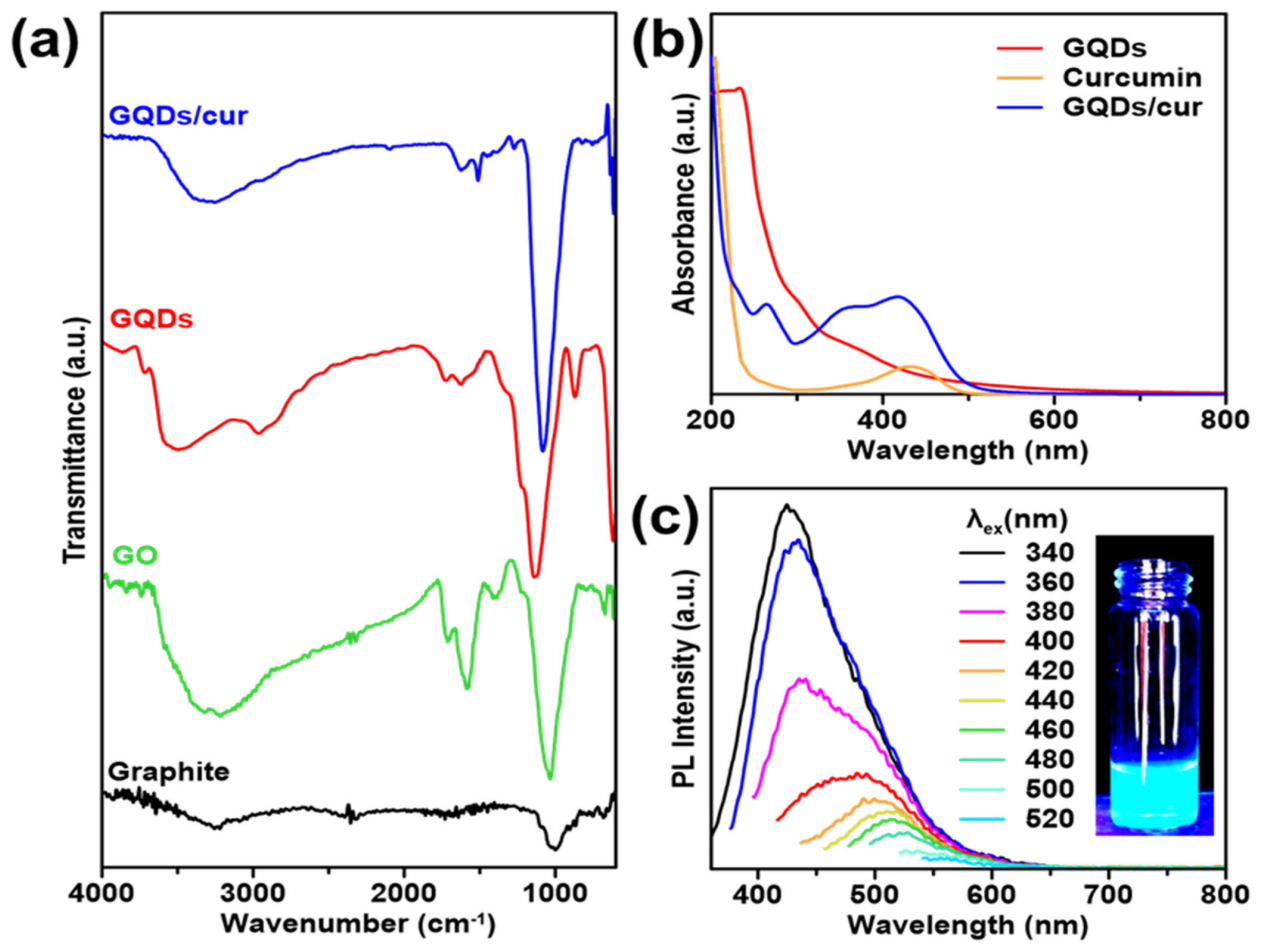
Chemical bonding interaction and optical properties of GQDs. (a) FTIR transmittances of bare graphite, GO, GQDs, and as-product GQDs/cur. (b) UV-vis spectra of GQDs (red), curcumin (orange), and GQDs/cur (blue). (c) PL emission spectra of GQDs under varied excitation wavelength source (340 - 520 nm), equipped with fluorescence image of GQDs under 365 nm UV light.

FTIR spectral studies were undertaken to investigate the interaction between GO and GQDS. FTIR analysis enables the identification of functional groups and molecular impurities on nanoparticle surfaces. The FTIR spectrum of the GQDs is shown in **Figure 2a**, which illustrates that the graphite contains no absorption bands in any group since it only consists of carbon. In the GO spectra, an absorption band at wave number 3364 cm^-1^ is attributed to the stretching vibration of the -OH group. Another spectrum at wave number 1720 cm^-1^ shows the vibration of C=O stretching. The peak at wave number 1628 cm^-1^ indicates the vibration of C=C stretching. Additionally, a peak at wave number 1030 cm^-1^ suggests the presence of C-O bonds. In the case of GO spectra, the -OH group can be observed at 3410 cm^-1^, the C=O stretching group at 1743 cm^-1^, C=C stretching at 1637 cm^-1^, and the C-O bond at a wave number of 1054 cm^-1^ 32, 33. The GQDS spectra show a strong absorption band of hydrophilic groups, including the -OH group at 3470 cm^-1^ and the carboxylic -OH group at 2959 cm^-1^. The presence of polar groups, such as hydroxyl and carbonyl, can be indicated by a hydrophilic compound 26. It can be observed that GO and GQDs have quite similar spectra. Both materials display an absorption band at around 1440-1625 cm^-1^ related to the aromatic C=C group, which indicates that GQDs have been successfully synthesized 8, 9, 34. The presence of the aromatic C=C group is a distinguishing characteristic that confirms the existence of the GQDs structure. The spectra of GQDs/cur showed the -OH absorption band at 3257 cm^-1^ and the aromatic C=C group at 1512 cm^-1^. The compounds' polarity proves the presence of GQDs, and their aromatic groups allow successful binding to curcumin, that could be demonstrated through the decrement of -OH transmittance level of GQDs/cur compared with as-synthesized GQDs.

The optical properties of the synthesized materials were investigated through the UV-absorption and photoluminescence. The absorption wavelengths of GQDs, curcumin, and GQDs/cur samples were determined using a UV-Vis Spectrophotometer. From the UV-Vis image in **Figure 2b**, it is evident that the GQDs spectra exhibit two peaks: a maximum absorbance peak and a shoulder peak. The GQDs sample exhibits maximum absorbance at 299 nm, indicating the transition of *π* → π* electrons in sp^2^-hybridized aromatic system 35. Although the shoulder peak of GQDs can be observed at a wavelength of 366 nm, it is believed that the transition of unbonded electrons to the bonding core orbital (n → π*) is caused by a C=O group. This absorption occurs at a longer wavelength and has a lower intensity. These findings suggest that the GQDs contain C=O functional groups and aromatic bonds. In the spectra of curcumin, the maximum absorbance occurs at 432 nm, which is consistent with previous studies 36. The spectra of GQDs/cur exhibit three peaks at wavelengths of 263 nm, 369 nm, and 415 nm. Two of these peaks are approximately the same wavelength as curcumin and GQDs but have lower absorbance due to the shift in absorbance peak. This indicates that GQDs and curcumin were successfully loaded, as evidenced by the peak of GQDs and curcumin in the third spectrum. In addition,** Figure 2c** displays the PL spectrum of the synthesized GQDs, which exhibit luminescent properties as evidenced by their emission intensity peak. This strong evidence supports quantum dot formation as blue luminescence corresponds to a 340 nm max emission wavelength. As the intensity of the blue color increases, the size of the GQDs produced decreases 34. The PL spectra of the GQDs sample show that the GQDs sample has excitation-dependant properties at emission wavelengths of 320 nm to 400 nm. The spectrofluorometer analysis results also show that the GQDs sample's quantum yield is 1.32%. The GQDs sample exhibits a high fluorescence quantum yield, indicating potential for use in drug delivery due to its favorable optical properties.

### Curcumin release profile analysis

**Figure 3:**
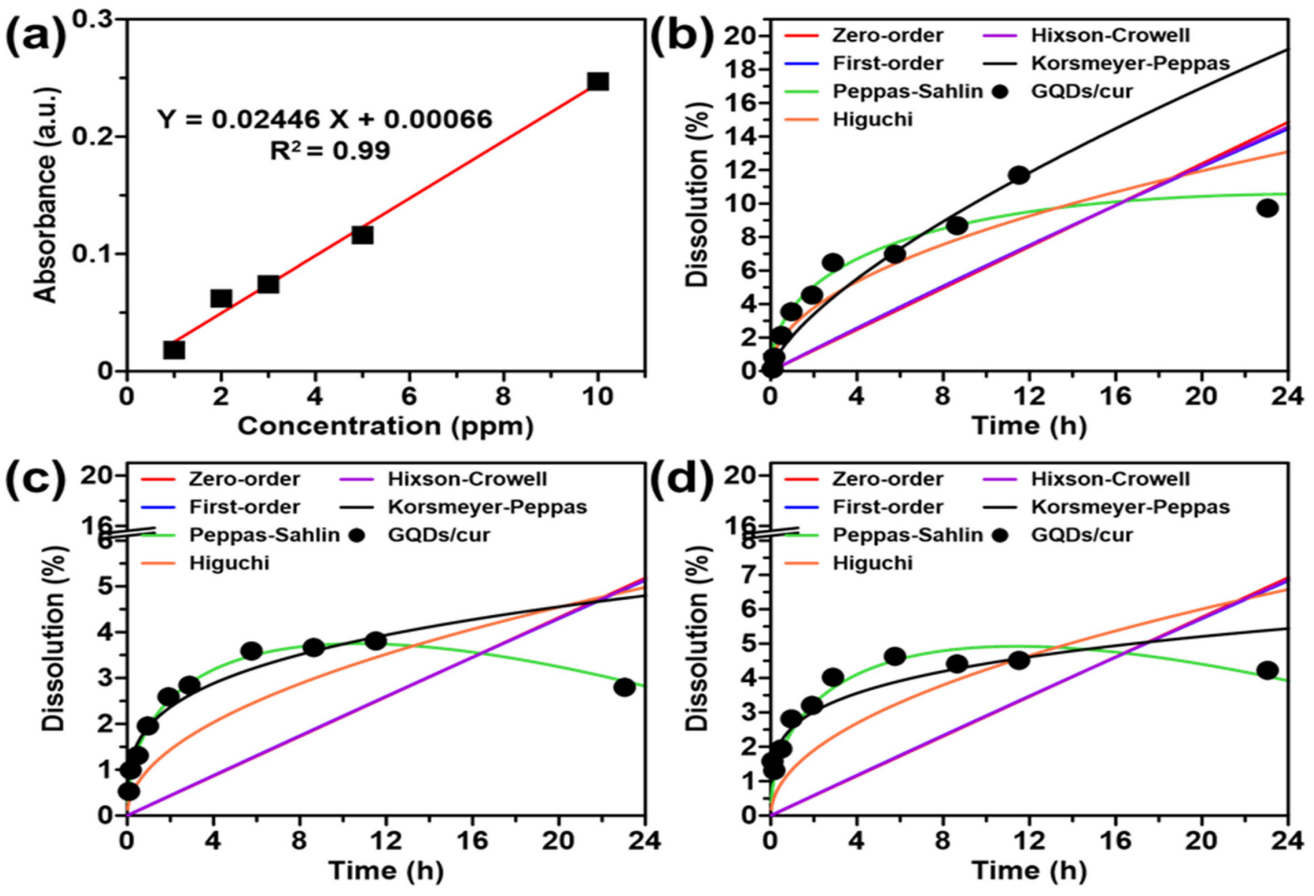
(a) Calibration curve of curcumin. Kinetical release of GQDs/cur using varied mathematical models, including 0-order, 1-order, Peppas-Sahlin, Higuchi, Hixson Crowell, and Korsmeyer-Peppas under (b) pH 4, (c) pH 7, and (d) pH 9.

The drug release kinetics estimates the drug concentration emitted by various release models. The drug release profile was conducted with nine drug release kinetics modeling, namely zero-order, first-order, Korsmeyer-Peppas T-lag, Higuchi's, Hopfenberg, Hixson-Crowell, Peppas-Sahlin, Baker Lonsdale, and Weibull models release kinetics 37. The six kinetics models were used to study the mechanism and rate of release of curcumin from GQDs/cur. At pH variations of 4, 7, and 9, the release of curcumin from GQDs/cur was observed, with data collected at 5, 10, 30, 60, 120, 180, 360, 540, 720, and 1440 minutes. **Figures 3b-d** illustrates the kinetics of curcumin release from GQDs/cur. The parameters in **Table S1** play a significant role in the curcumin release kinetics model, as can be seen by examining the model's predictions. The observed trend indicates that the samples adhere to the kinetics of the Peppas-Sahlin model at different pH values (4, 7, and 9) and corroborated by the Peppas-Sahlin model having the highest R^2^ value **(Figure 3a)** and the smallest chi-square (Ꭓ^2^) value across all pH ranges, indicating that the latter is accurate. The Peppas-Sahlin model explains the mechanism of drug release that occurs through coupling between the phenomenon of Fickian diffusion and the transition of the polymer from a semi-rigid state to a flexible state that results in relaxation of the polymer chain.

**Table S1** demonstrates that the value of K_1_ (positive) is greater than that of K_2_ (negative), indicating that the Fickian diffusion mechanism is more dominant than polymer chain relaxation. The Fickian diffusion mechanism is where the diffusion speed is slower than the relaxation speed of the matrix. The outcomes of this study are comparable to the release of curcumin diethyl disuccinate (CDD) from chitosan/alginate nanoparticles (CANPs), which also follows the Peppas-Sahlin kinetic model 38. A comparison of the Peppas-Sahlin *Κ*1 values at various pH levels revealed the highest release rate in an acidic environment (pH 4). It was concluded that the curcumin release rate using the Peppas-Sahlin kinetic model was 180% faster in an acidic environment than at neutral pH (7) and 141% faster than at alkaline pH (9). This increased release rate is likely due to the aggregation of GQDs in alkaline conditions during the colloidal stability test, which leads to the deposition of curcumin and reduces its release rate.

### Colloidal Stability Evaluation

**Figure 4:**
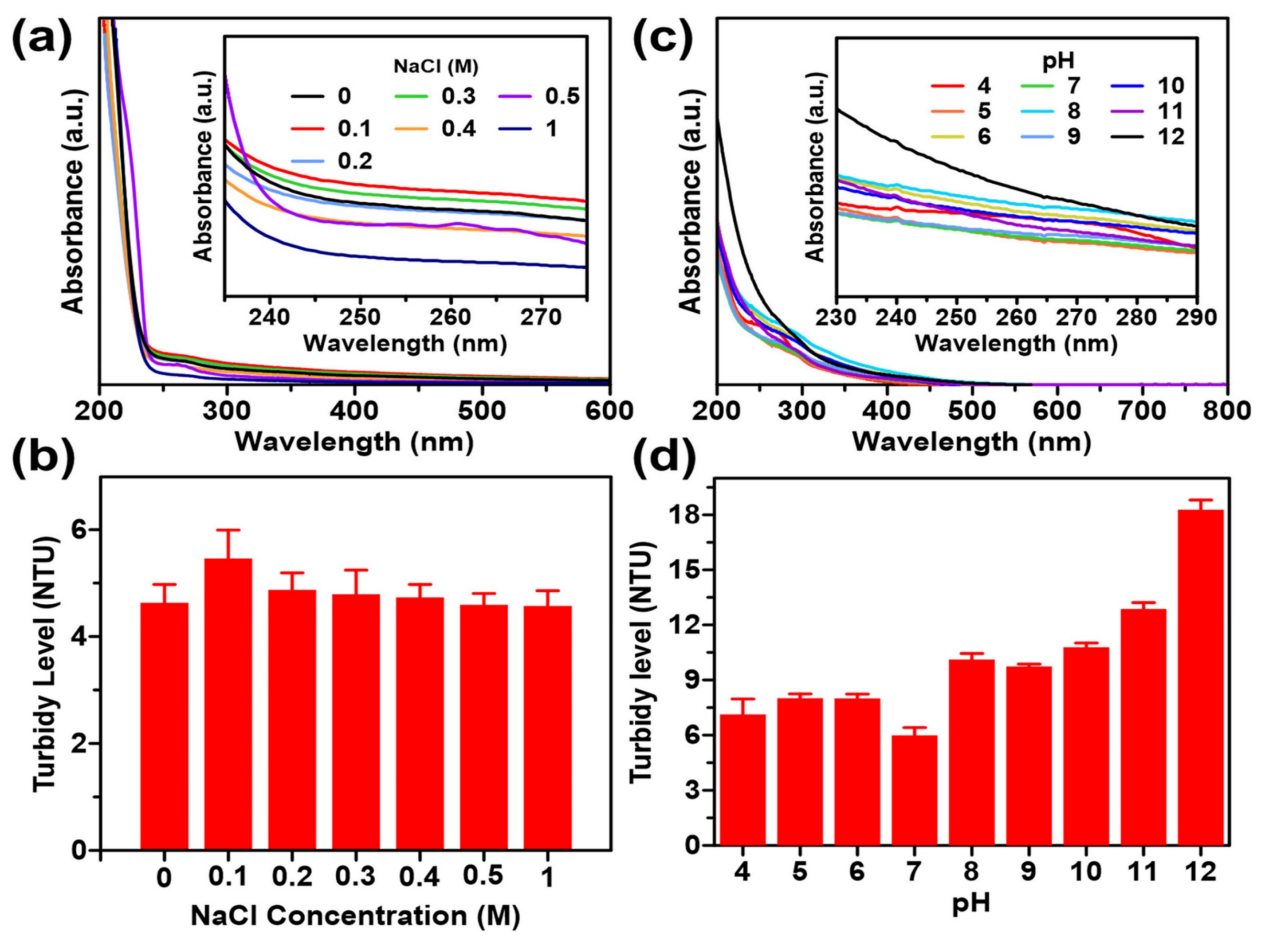
Colloidal stability of GQDs/cur using UV-Vis spectroscopy and turbidy test under varied (a, b) pH, (c, d) NaCl concentration.

Colloidal stability tests on GQDs with pH variation aim to determine the stability of GQDs in acidic to alkaline environments. The results indicated that the pH of the small intestine in each section ranges from 4.2-5.7 in the duodenum section, 5-6 in the jejunum section, and 5.8-6 in the ileum section. **Figure 4d** displays the results of the stability test analysis with pH variations in the range of 4 to 12. GQDs showed aggregation at pH 8-12 at hour 12 and precipitation at pH 10-12 at hour 24. **Figure 4c** demonstrates that after incubation for 24 hours, samples with a pH range of 8-12 have a greater absorbance than samples with a pH range of 4-7 because the development of aggregates causes the solution to be more brownish. Such stability is meritorious for substances that would be considered for application in body conditions 39.

**Figure S1** demonstrates that various concentrations of NaCl at varying times (0, 3, 6, 12, and 24 hours) have no discernible effect on the rate of colour degradation or precipitation. **Figure 4a** demonstrates no significant difference in response to an increment of NaCl concentration. Similarly, the effects on turbidity in **Figure 4b** do not indicate a substantial change in the stability of GQDs. This observation is bolstered by visual inspection, which demonstrated the absence of sediment or aggregates during the evaluation (as depicted in **Figure S1**). Consequently, GQDs exhibit relative stability within the range of salt concentrations differing from 0 to 1 M. For a substance to be appropriate for use in bioimaging, it must possess pharmacological properties compatible with the human body and be in nanoparticle form to avoid interference with cellular functions.

### *In vivo* performance

**Figure 5:**
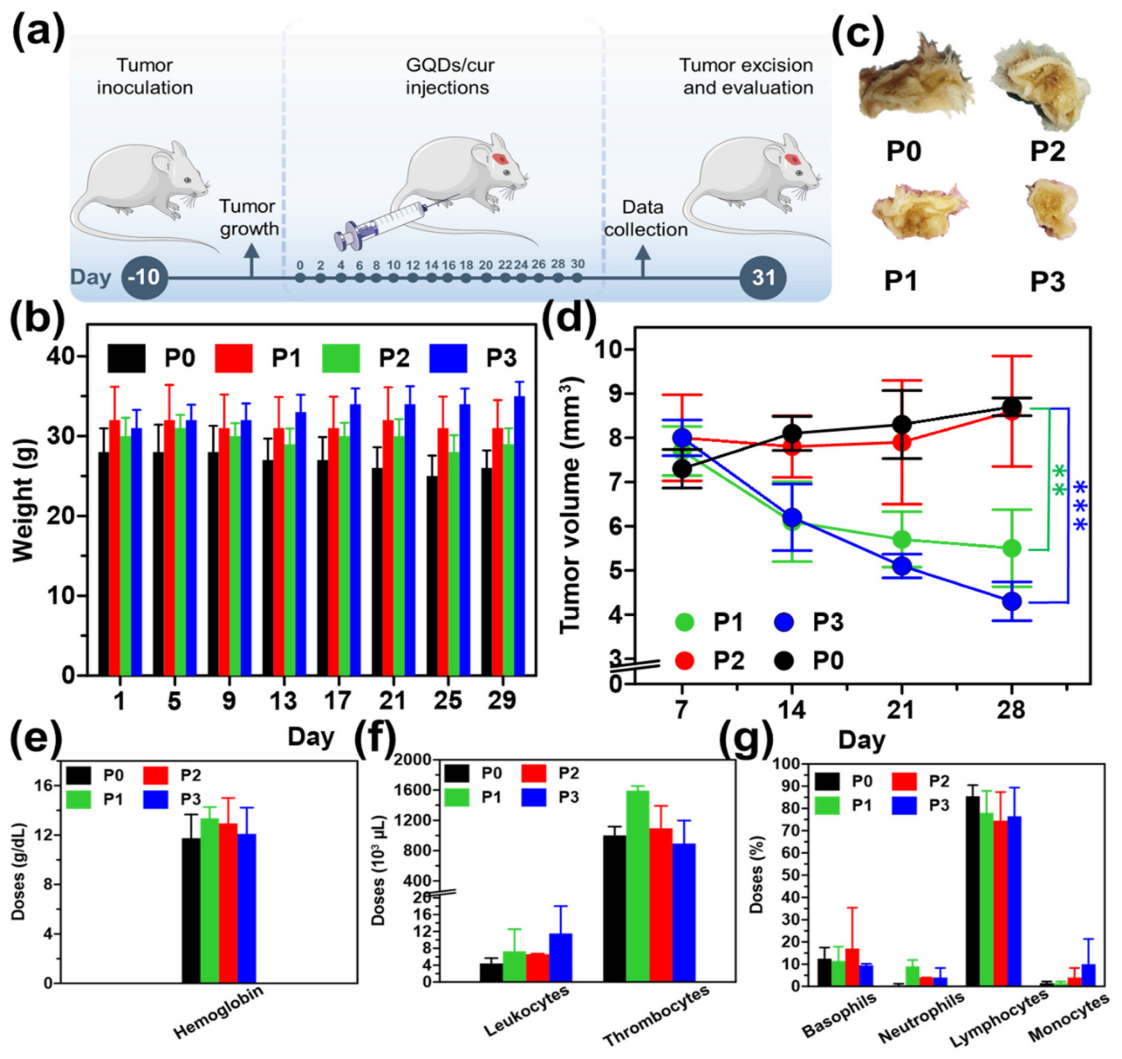
(a) Schematic illustration for the mice treatment experiment (dose) injected every two days for one months. (b) Body weight alteration of saline solution (P0), curcumin (P1), GQDs (P2), and GQDs/cur (P3). (c) Visual photographs of P0, P1, P2, and P3 post-treatment. (d) Tumor volume of the mice within 29 days observation. Histograms of the blood test results for each parameter (e) hemoglobin, (f) leukocytes, thrombocytes, (g) basophils, neutrophils, and lymphocytes. Each group consisted of 16 mice. The injection route was intraperitoneal, and tumor was a chemically induced model. The statistical study was conducted using a t-test. * refers a statistical significance (*p < 0.05, **p < 0.01 and ***p < 0.001) between the experimental data.

As experimental animals, white male mice at the age of eight weeks were applied in this study. Before receiving sample treatment, experimental animals were injected with the carcinogenic compound (benzopyrene) to establish a parameter.^40^ A benzopyrene induction period of ten days was observed. After that, 64 mice were subsequently divided into four treatment groups: each group received 30 mg/10 mL of benzopyrene dissolved in olive oil (*Oleum olivarum*). The following process was sample injection into mice every two days for approximately one month (**Figure 5a**). The injection method for each treatment group was through intraperitoneal (IP). The treatment group itself consisted of 4 groups. The first group, the P0 group, received saline solution (NaCl with a concentration of 0.9%). The mice were consistently injected every two days, with a volume of 0.2 mL per injection. During the initial fortnight following administration, the mice exhibited sustained activity and a sufficiently robust appetite. However, during the final two weeks, a subset of the mice demonstrated signs of sedentary behavior, decreased appetite, as well as fur flaking 40-44. Treatment group P1 received curcumin for one month. No observable alterations in the mice's behavior were observed, and their fur and appetite exhibited generally favorable conditions. However, the nodule size reduction observed in mice was not particularly noticeable. GQDs in the P2 treatment group developed virtually identically to those in the P0 treatment group. The physical condition of the mice deteriorated significantly over time, as evidenced by their decreased activity, appetite, and overall health. Lastly, the P3 treatment group, GQDs/cur, exhibited generally favorable conditions among the mice. Most of the mice remain physically fit, have a healthy appetite, and are in excellent condition, except for their fur and scars caused by protruding cancer that are beginning to heal gradually. In the P3 group, the diameter of the malignancy also decreased considerably (Depicted in Supplementary **Figure S2**).

Analysis of the body weight of the mice revealed that in the P0 and P2 groups, a gradual decline in body weight was observed. This is due to the elevated stress levels induced by cancer and the subsequent reduction in appetite (**Figure 5b**). Comparatively, the P1 group exhibits a fluctuating pattern of stability. The mice in the P3 group experienced a gradual increase in body weight as a result of the decreased impact of cancer. As a consequence, the mice experienced a decrease in pain and tension levels, which was followed by an enhancement in their appetite. In addition to monitoring the body weight, psychological change was also assessed. More specifically, a decline in activity levels was observed among participants in the P0 and P2 groups on day seventeen, transitioning from a state of active movement to one of increased silence. In addition, hair loss was observed in groups P0 and P2, suggesting that the mice were under a great deal of stress; some mice in the treatment group even perished. In contrast, mice assigned to groups P1 and P3 maintained healthy fur throughout the duration of the observation and exhibited a propensity for active movement. Continuing observations of both mice's body weight and measuring cancer volume are also parameters to determine the effectiveness of the as-synthesized GQDs/cur tested as drug delivery. The measurement of the diameter of the cancer itself is examined once a week for one month. In general, as depicted in **Figures 5c-d**, groups P0 and P2 continue to undergo substantial enlargement due to the ineffectiveness of the injected substance in inhibiting cancer growth; consequently, the cancer will persist in its progression until ultimately fatal. In contrast, the malignancy diameter exhibited a reduction in both the P1 and P3 treatment groups, with the P3 treatment group demonstrating the most substantial shrinkage (50.6% compared to P0) due to the two injected substances that can impede the proliferation of cancer in mice. The loading of GQDs/cur in the P3 treatment group decreased more rapidly than in the P1 treatment group, which received curcumin alone via injection. As a result, GQDs may be a potential candidate for drug delivery systems.

The hemoglobin levels in all four treatments are within the expected range (Supplementary **Table S2**), according to the findings of a complete blood count, particularly hematology (demonstrated in **Figure 5e**). On the other hand, leukocyte levels in group P3 were marginally higher than that of P0, P1, and P2 groups thus indicating a triggered immune response due to the treatment. Due to disorders or diseases that stimulated excessive platelet formation in the organs of the mice, thrombocyte doses in the four groups of mice subsequently increased by a substantial margin, surpassing normal levels (**Figure 5f**). The elevated concentrations of basophils in the four mice are attributed to their function in preventing blood clot formation and combating parasites as well as responding to a stressor within the body. This function is compromised due to the immune system's assault on healthy cells or body tissue, which provides evidence of disease (tumor) in mice. In all treatment groups, neutrophil levels in mice were below normal; this condition renders the mice's bodies incapable of combating pathogenic microbes, rendering them susceptible to disease. Furthermore, the reduction in neutrophil counts can be attributed to the adverse effects associated with chemotherapy. Subsequently, lymphocytes contribute to the maintenance of the body's immune system by combating pathogens and toxins; however, only group P2 exhibited normal lymphocyte counts. Curcumin administration in other groups results in the elevation of lymphocyte counts. Contrarily, the P0 group experiences inflammation due to the direct effects of benzopyrene. Monocyte counts in mice assigned to group P3 were elevated compared to those in groups P0, P1, and P2. The observed discrepancy in the findings can be attributed to the active defense mechanism exhibited by the mice in group P3 against infection or disease. The final parameter, blood sedimentation rate, remained within normal limits for all treatment groups, indicating that none of the treatments impacted the blood sedimentation rate in mice.

The results from the comprehensive blood test indicate that the albumin levels in groups P0, P1, and P3 exhibit a minor deviation below the established normal range, which is associated with swelling resulting from compromised blood circulation (as shown in Supplementary **Table S3**). Subsequently, SGOT (Serum Glutamic Oxaloacetic) levels in mice from the four groups experienced a significant increase that exceeded normal levels, which was caused by increased metabolism due to the effects of benzopyrene, thereby increasing the liver's workload. SGOT is an enzyme in the liver whose role is to digest protein 45, 46. However, if the increase in SGOT is does not triple its normal level, then it is still within reasonable limits and will not cause dangerous or fatal effects. SGPT (Serum Glutamic Pyruvic Transaminase) is an enzyme also contained in the liver which plays a role in digesting protein in the body 45-47. Except for the P3 treatment group, which was marginally above normal, all other groups exhibited levels within the normal range. This data suggests that benzopyrene exerts an effect that induces hepatic fat accumulation. P0, P1, and P2 were at normal levels in the gamma GT groups, whereas group P3 was marginally above the average dose, but still within acceptable limits. The total bilirubin concentrations of the four treatments remained within the expected range, as the treatments to the mice did not impact the bilirubin's functionality in the mice's blood. The BUN (Blood Urea Nitrogen) concentrations in the P0 and P3 treatment groups subsequently returned to normal, whereas P1 and P2 groups experienced an increment above average. The mice consumed very little water, consequently, it results in elevated blood urea levels. Creatinine levels in all four treatment groups show below the established dosage limit, which can be attributed to the dehydration of the mice. Low levels of creatinine impair mice's activity and muscle function 48, 49.

## Discussion

### Curcumin Release Profile Analysis

The investigation into the release kinetics of curcumin from GQDs/cur involved a comprehensive analysis using nine different release models. The study focused on pH variations of 4, 7, and 9, with data collected at multiple time points. Notably, the Peppas-Sahlin model emerged as the most fitting, as evidenced by its highest R^2^ value and the smallest chi-square value across all pH ranges. The Peppas-Sahlin model provides insights into the mechanism of drug release, involving a coupling between Fickian diffusion and the transition of the polymer from a semi-rigid to a flexible state, resulting in polymer chain relaxation. The analysis of K1 and K2 values in **Table S1** indicates that Fickian diffusion dominates over polymer chain relaxation, suggesting a slower diffusion speed compared to relaxation speed. Interestingly, this aligns with the release kinetics observed in the study of curcumin diethyl disuccinate (CDD) from chitosan/alginate nanoparticles (CANPs), where the Peppas-Sahlin model was also applicable. Moreover, the study delved into the impact of pH on curcumin release rates, revealing that an acidic environment (pH 4) led to a kinetic rate 180% faster than pH 7 and 141% faster than pH 9. This finding underscores the importance of considering environmental factors in optimizing drug release formulations.

### Colloidal Stability Evaluation

The colloidal stability tests on GQDs with variable pH were designed to evaluate their stability in conditions similar to various parts of the small intestine. The results indicated a pH-dependent aggregation pattern, with GQDs showing aggregation at pH 8-12 at hour 12 and precipitation at pH 10-12 at hour 24. More importantly, the stability test results were aligned with the pH variations observed in the small intestine sections. The study found that GQDs exhibited relative stability within the pH range of 4 to 7, demonstrating a promising characteristic for potential biological applications. This stability is crucial, as it suggests that GQDs could withstand the physiological conditions of the gastrointestinal tract. Besides, the evaluation of NaCl concentration effects on GQD stability indicated no discernible impact on color degradation or precipitation, emphasizing the robust stability of GQDs across various salt concentrations. Given their stability within the human body and compatibility with cellular functions, both of which are crucial prerequisites, this attribute establishes GQDs as promising candidates for biomedical applications. Overall, the release kinetics and colloidal stability assessments provide valuable insights into the potential efficacy and applicability of GQDs/cur as a drug delivery system for curcumin.

### Tumor Inhibition Performance

The findings underscore the promising potential of the combined treatment involving Graphene Quantum Dots (GQDs) and curcumin in the P3 group. The most striking revelation lies in the substantial reduction in tumor size observed within this group. This outcome strongly suggests a synergistic effect between GQDs and curcumin, showcasing their combined efficacy in impeding the growth of cancer cells. The intricate interplay between these two components seems to yield a more potent anti-cancer effect than when administered individually, marking a significant stride in the quest for advanced and more effective cancer treatments.

### Body Weight and Psychological Well-being

The well-being of the mice in the P3 group stands out prominently, reflecting the broader impact of the GQDs/cur treatment on overall health. Not only did these mice experience an increase in body weight, signalling a positive shift away from the stress-induced decline seen in other groups, but they also displayed reduced psychological stress. This dual improvement indicates that the GQDs/cur regimen not only targets the physical manifestation of cancer but also has a positive influence on the psychological well-being of the subjects. Such holistic benefits are crucial in understanding the potential of this treatment to enhance the quality of life for cancer patients.

### Hematological and Blood Test Findings

An essential aspect of evaluating any treatment's viability is its impact on hematological parameters. Encouragingly, the study reveals that the GQDs and curcumin treatments maintained hematological parameters within the expected ranges, suggesting a lack of adverse effects on blood composition. The observed elevation in leukocyte and thrombocyte levels in response to treatment implies an active immune system response. While this response may be influenced by the presence of cancer and the administered treatments, it further underscores the complexity of the immune system's involvement in cancer therapy.

Liver enzyme levels, particularly SGOT and SGPT, increased in response to the carcinogenic benzopyrene. Importantly, these increases remained within acceptable limits, indicating that the hepatic stress induced by the experimental conditions was manageable. This finding provides reassurance regarding the safety of the treatment, considering the potential side effects on liver function commonly associated with cancer therapies.

### Implications for Curcumin Delivery

The study's implications for drug delivery are significant and open new avenues for therapeutic interventions. The utilization of GQDs and curcumin showcases a potential strategy for effective drug delivery systems. The observed synergy between these components suggests that GQDs may enhance the delivery and efficacy of therapeutic agents like curcumin. This insight could pave the way for the development of more targeted and efficient drug delivery systems in cancer treatment, minimizing side effects and maximizing the therapeutic impact.

### Renal and Muscular Function

The examination of renal and muscular function introduces a note of caution into the overall optimism. Elevated Blood Urea Nitrogen (BUN) levels, indicative of dehydration, raise concerns about potential impacts on renal function. Additional research is necessary to comprehend the implications of the correlation between BUN levels and renal health. Furthermore, a comprehensive investigation into the impact of the treatment on the musculoskeletal system is warranted in light of the observed low creatinine levels, which are correlated with compromised activity and muscle function. This dual consideration of renal and muscular function underlines the necessity for comprehensive assessments when developing and implementing novel cancer therapies.

## Conclusion

In conclusion, the comprehensive characterization of graphene quantum dots (GQDs) synthesized through a hydrothermal process involving graphite oxidation and subsequent functionalization has provided valuable insights into their structural and optical properties. Various analytical techniques such as XRD, AFM, Raman spectroscopy, and FTIR have elucidated the nanoparticle-sized nature of GQDs, their crystalline structure, and the presence of functional groups. The homogeneity of particle size, as determined by AFM, revealed an average diameter of approximately 6.44 ± 0.05 nm, confirming the successful synthesis of GQDs. The optical properties, assessed through UV-PL studies, demonstrated the quantum dot formation with a blue luminescence peak. In addition, the release kinetics of curcumin from GQDs, fitted by the Peppas-Sahlin equation, revealed the highest release rate in acidic conditions (pH 4). The *in vivo* performance of GQDs/curcumin was evaluated using a murine model injected with a carcinogenic compound. The treatment exhibited promising results in inhibiting tumor growth, with the GQDs/curcumin group (P3) showing a substantial reduction (50.6% compared to P0) in malignancy diameter compared to other groups. The observations included improvements in overall health, body weight, and fur condition, highlighting the potential therapeutic efficacy of GQDs as drug carriers. Additionally, the analysis of blood parameters and organ function indicated that GQDs/curcumin treatment had minimal adverse effects, suggesting its biocompatibility and potential for further biomedical applications. Overall, these findings emphasize the promising potential of GQDs as efficient carriers for the targeted delivery of the curcumin cancer drug.

## Supplementary Material

Supplementary figures and tables.
