# Supplementary material for: Unraveling In vivo Potential of Curcumin-loaded Graphene Quantum Dots on Drug Delivery and Release Kinetics Aspects of Cancer Treatment
Source: Nanotheranostics. 2024 Aug 13;8(4):521–34. doi: 10.7150/ntno.96559 (PMC11539179; doi:10.7150/ntno.96559)
Supplement: Supplementary file 1 — Supplementary figures and tables. [file ntnov08p0521s1.pdf]

Supporting Information

Unraveling In Vivo Potential of Curcumin-loaded Graphene Quantum Dots on Drug Delivery and Release Kinetics Aspects of Cancer Treatment

Siti F. A. Sugito,[a,b,1] and Aswandi Wibrianto,[a,b,1] Siska Novania,[a] Shinta Widyastuti,[a] Musbahu Adam Ahmad,[a] Satya C. W. Sakti,[a,b] Lee H. Voon[c] and Mochamad Z. Fahmi,[a,b]*

a. Department of Chemistry, University Airlangga, Surabaya 60115, Indonesia

b. Supra Modification Nano-Micro Engineering Research Group, Universitas Airlangga, Surabaya 60115, Indonesia

c. Nanotechnology and Catalysis Research Centre (NANOCAT), Universiti Malaya, 50603, Kuala Lumpur, Malaysia

[1]Siti F. A. Sugito and Aswandi Wibrianto contributed equally to this work

***Corresponding Author**

Mochamad Zakki Fahmi

Department of Chemistry, University Airlangga, Surabaya 61115, Indonesia

Supra Modification Nano-Micro Engineering Research Group, Universitas Airlangga, Surabaya 60115, Indonesia

E-mail: m.zakki.fahmi@fst.unair.ac.id

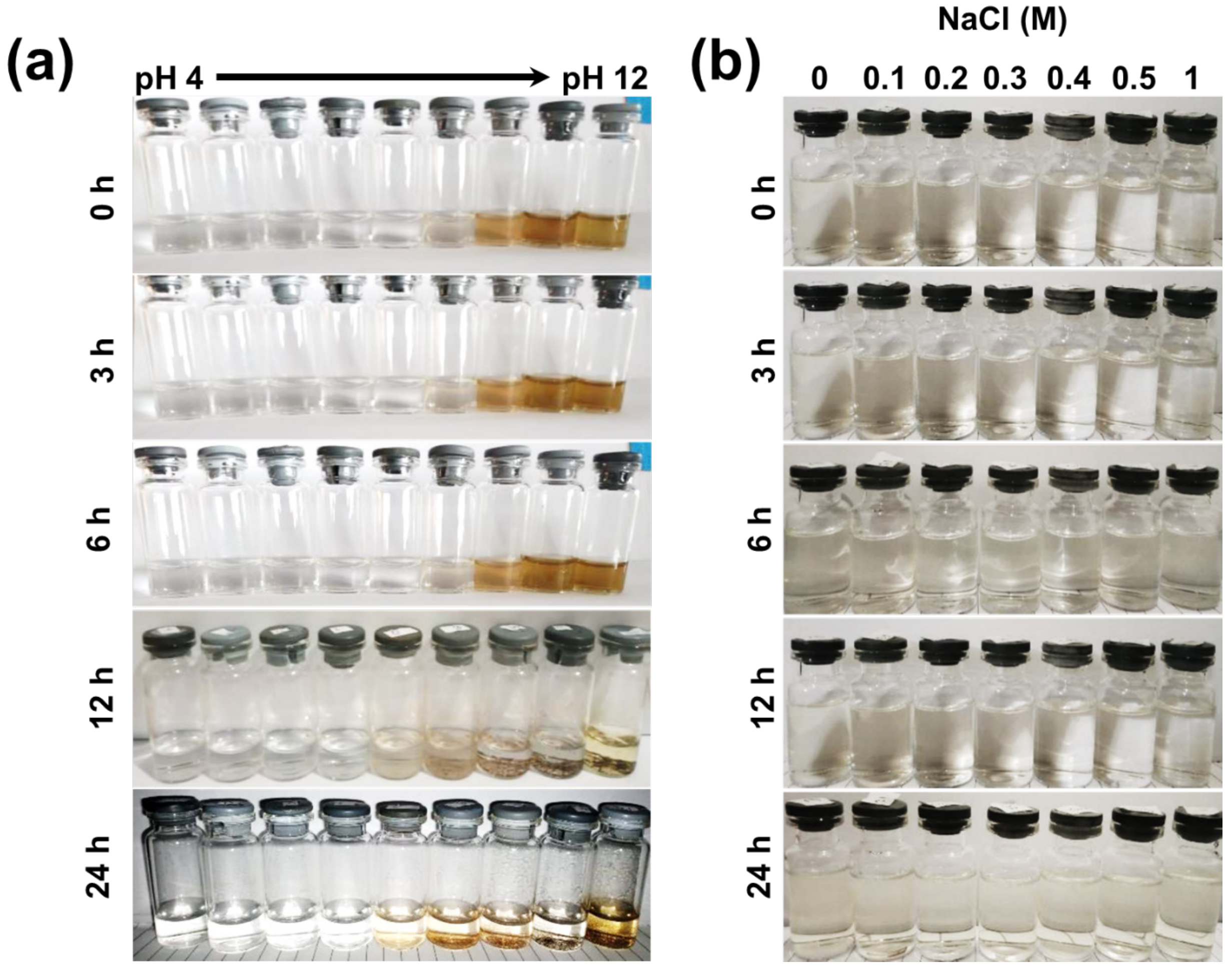

Figure S1. Photograph of GQDs colloidal stability under varying pH (1-12) and NaCl concentration (0-0.5 M) for 24 h incubation.

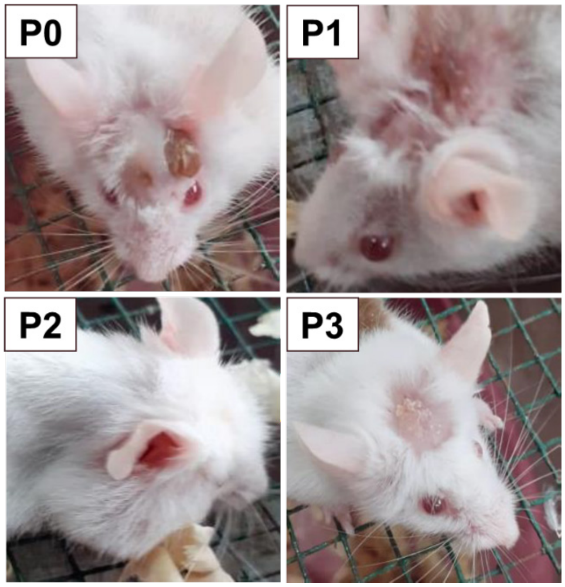

Figure S2. Visual image of tumor to mice under different treatments after 29 days observation (P0, negative control; P1, curcumin; P2, GQDs; P3, GQDs/cur).

Table S1. The parameters of several mathematical models that influence the drug release in GQDs/cur.

Formula	Parameter	Zero-order	First-order	Peppas-Sahlin	Higuchi	Hixson-Crowell	Korsmeyer-Peppas
GQDs/cur pH 4	M_0 (g)	0,02	0,02	0,02	0,02	0,02	0,02
	χ^2	58,6621	54,008	2,2365	3,4206	55,8641	7,8389
	R^2	0,6039	0,6516	0,9514	0,8482	0,6448	0,7642
	K_1	6,6039	0,0001	0,5377	0,3449	0,000,	0,1204
	K_2	-	-	0,0068	-	-	-
	n	-	-	-	-	-	0,6974
GQDs/cur pH 7	M_0 (g)	0,02	0,02	0,02	0,02	0,02	0,02
	χ^2	92,4467	90,6594	0,0529	5,5092	91,2762	1,1844
	R^2	0,3167	0,3234	0,9948	0,5883	0,3212	0,7237
	K_1	0,0035	0,0001	0,2958	0,1311	0,0001	0,6066
	K_2	-	-	-0,0052	-	-	-
	n	-	-	-	-	-	0,2842
GQDs/cur pH 9	M_0 (g)	0,02	0,02	0,02	0,02	0,02	0,02
	χ^2	209,153	204,1898	0,9448	10,9181	205,8312	0,7109
	R^2	0,4236	0,424	0,9619	0,6853	0,4295	0,8258
	K_1	0,0048	0,0001	0,3763	0,1733	0,0001	0,9712
	K_2	-	-	-0,0073	-	-	-
	n	-	-	-	-	-	0,2368

Table S2. Blood parameter evaluation of GQDs/cur.

Blood parameter	Normal doses	P0	P1	P2	P3	P-value[1]	P-value[2]	P-value[3]
Hemoglobin (g/dL)	11.6 – 16.1	11.75 ± 1.91	11.2 ± 1.70	11.95 ± 0.21	12.1 ± 2.12	0.39475	0.45347	0.43914
Leukocytes (10^3 µL)	2.0 – 10.0	4.4 ± 1.27	7.3 ± 5.23	6.6 ± 0.14	11.5 ± 6.51	0.29282	0.12429	0.18573
Thrombocytes (10^3 µL)	150.0 – 450.0	1002.5 ± 113.84	1593.5 ± 61.52	1096 ± 295.57	893.5 ± 304.76	0.01157	0.37412	0.35915
Basophils (%)	0.5 – 1.0	12.5 ± 4.95	11.5 ± 6.36	17 ± 18.38	9.5 ± 0.71	0.43845	0.39732	0.27602
Neutrophils (%)	12.0 – 38.0	0.5 ± 0.71	9 ± 2.83	4 ± 0	4 ± 4.24	0.07574	0.04517	0.22772
Lymphocytes (%)	60.0 – 75.0	85.5 ± 4.95	78 ± 9.90	74.5 ±14.85	76.5 ± 14.85	0.25677	0.25098	0.28269
Monocytes (%)	1.0 – 6.0	1.5 ± 0.71	1.5 ± 0.71	4 ± 4.24	10 ± 11.31	0.50000	0.28100	0.24067
Erythrocyte sedimentation rate (ESR) (mm)	0 – 2	2 ± 0	2 ± 0	2 ± 0	–	–	–	

P-value[1]: P-value of P0 and P1; P-value[2]: P-value of P0 and P2; P-value[3]: P-value of P0 and P3

Table S3. Blood parameter evaluation of GQDs/cur.

Blood parameter	Normal doses	P0	P1	P2	P3	P-value[1]	P-value[2]	P-value[3]
Albumin (g/dL)	3.0 – 5.0	2.8± 0.14	2.9± 0.14	3±0	2.7± 0.14	0.276	0.148	0.276
SGOT (U/L)	23.2 – 48.4	160.5 ±0.71	121.5 ± 20.51	116.5 ±6.36	112.5 ±6.36	0.113	0.033	0.030
SGPT (U/L)	2.1 – 23.8	45.5 ±34.65	38.5 ±9.19	37.5 ±9.19	17.5± 4.95	0.414	0.403	0.230
Gamma GT (U/L)	0 – 30	16±0	13.5 ±2.12	12±0	30.5± 2.12	0.172	-	0.033
Total Bilirubin	0 – 0.55	0.210 ±0.03	0.29	0.16	0.22	0.213	0.121	0.450
BUN	8.92 – 17.89	14.75	21.25	30	15.25	0.225	0.194	0.429
Creatinine (mg/dL)	0.30 – 1.00	0.03	0.065	0.115	0.115	0.209	0.042	0.042

P-value[1]: P-value of P0 and P1; P-value[2]: P-value of P0 and P2; P-value[3]: P-value of P0 and P3
